# Multiple Frequency Bands Analysis of Large Scale Intrinsic Brain Networks and Its Application in Schizotypal Personality Disorder

**DOI:** 10.3389/fncom.2018.00064

**Published:** 2018-08-03

**Authors:** Shouliang Qi, Qingjun Gao, Jing Shen, Yueyang Teng, Xuan Xie, Yueji Sun, Jianlin Wu

**Affiliations:** ^1^Sino-Dutch Biomedical and Information Engineering School, Northeastern University, Shenyang, China; ^2^Department of Radiology, Affiliated Zhongshan Hospital of Dalian University, Dalian, China; ^3^Department of Psychiatry and Behavioral Sciences, Dalian Medical University, Dalian, China

**Keywords:** intrinsic brain network, resting sate fMRI, power spectral density, low-frequency fluctuation, schizotypal personality disorder

## Abstract

The human brain is a complex system composed by several large scale intrinsic networks with distinct functions. The low frequency oscillation (LFO) signal of blood oxygen level dependent (BOLD), measured through resting-state fMRI, reflects the spontaneous neural activity of these networks. We propose to characterize these networks by applying the multiple frequency bands analysis (MFBA) to the LFO time courses (TCs) resulted from the group independent component analysis (ICA). Specifically, seven networks, including the default model network (DMN), dorsal attention network (DAN), control executive network (CEN), salience network, sensorimotor network, visual network and limbic network, are identified. After the power spectral density (PSD) analysis, the amplitude of low frequency fluctuation (ALFF) and the fractional amplitude of low frequency fluctuation (fALFF) is determined in three bands: <0.1 Hz; slow-5; and slow-4. Moreover, the MFBA method is applied to reveal the frequency-dependent alternations of fALFF for seven networks in schizotypal personality disorder (SPD). It is found that seven networks can be divided into three categories: the advanced cognitive networks, primary sensorimotor networks and limbic networks, and their fALFF successively decreases in both slow-4 and slow-5 bands. Comparing to normal control group, the fALFF of DMN, DAN and CEN in SPD tends to be higher in slow-5 band, but lower in slow-4. Higher fALFF of sensorimotor and visual networks in slow-5, higher fALFF of limbic network in both bands have been observed for SPD group. The results of ALFF are consistent with those of fALFF. The proposed MFBA method may help distinguish networks or oscillators in the human brain, reveal subtle alternations of networks through locating their dominant frequency band, and present potential to interpret the neuropathology disruptions.

## Introduction

Resting state functional MRI (rs-fMRI) has been considered as a powerful tool in discovery science of human brain (Biswal et al., 2010; Buckner et al., 2013). Spontaneous low-frequency oscillations (LFOs) in the resting state blood oxygen level dependent (BOLD) signal can be acquired through rs-fMRI *in vivo* (Fox and Raichle, 2007), and the signals are thought to reflect spontaneous neuronal activity. Since Biswal et al. (1995) demonstrated that the signals in some spatially distributed brain regions were synchronized or correlated significantly in the motor system, many other brain systems have been discovered. These distributed brain regions construct different large-scale intrinsic connectivity networks (ICNs), each of which may correspond to one specific function (van den Heuvel and Hulshoff Pol, 2010; Raichle, 2011). Some representative, important and consistently reported ICNs include the default mode network (DMN), the dorsal attention network (DAN), the control executive network (CEN), the silence network, the somatomotor, visual and auditory networks.

There are at least two categories of data-driven approaches which have been widely employed to identify large-scale ICNs from resting-state fMRI data for further functional connectome analysis in time domain. The first category is the independent component analysis (ICA; Calhoun et al., 2001). Through ICA, fMRI data is decomposed into a summation of independent components (ICs), and each component contains a weighted set of voxels (i.e., the component’s spatial map) and a single time course (TC) that is common to those identified voxels (Beckmann and Smith, 2004). The second is to do clustering analysis based on voxel-wised rs-fMRI signals (Bellec et al., 2006; Lashkari et al., 2010; Blumensath et al., 2013; Thirion et al., 2014). For example, Yeo et al. (2011) identified seven coarse-grained ICNs and 17 fine-grained ICNs using clustering algorithm based on 1000 subjects’ rs-fMRI data from Human Connectome Project. Combination of these two methods (ICA and clustering method) might be beneficial, but has not been studied.

Analysis of rs-fMRI data in frequency domain is another important alternative to the temporal-spatial analysis. Zang et al. (2007) and Zou et al. (2008) proposed two Fast Fourier Transform (FFT) based indices of LFO amplitude: (1) amplitude of low frequency fluctuations (ALFF); (2) fractional amplitude of low frequency fluctuations (fALFF). ALFF indicates the power in the frequency band of 0.01–0.1 Hz, and fALFF is the power in 0.01–0.1 Hz divided by the total power in the entire detectable frequency range. Furthermore, Buzsaki and colleagues proposed the model that the power spectrum of neuronal oscillations forms a linear progression on the natural logarithmic scale, indicating that the oscillations can be separated into several independent frequency bands and each band might correspond a distinct oscillator with specific property and physiological function (Penttonen and Buzsaki, 2003; Buzsáki and Draguhn, 2004). Inspired by these observation, spontaneous LFOs in rs-fMRI have been decomposed into four frequency bands, slow-5 (0.01–0.027 Hz), slow-4 (0.027–0.073 Hz), slow-3 (0.073–0.198 Hz) and slow-2 (0.198–0.25 Hz; Zuo et al., 2010). It has been demonstrated that amplitudes of LFOs in slow-4 band were higher than that in slow-5 in some regions including the basal ganglia, thalamus and precuneus, whereas the opposite trend was found in lingual gyrus, middle temporal gyrus, inferior frontal gyrus and ventromedial frontal gyrus (Zuo et al., 2010; Han et al., 2011; Yu et al., 2014). Recently this approach has been employed to reveal the frequency-dependent ALFF alternation in neurological and psychiatry disorders, such as mild cognitive impairment (Han et al., 2011), epilepsy (Wang Z. et al., 2014; Wang L. et al., 2016), internet gaming disorder (Lin et al., 2015), social anxiety disorder (Zhang et al., 2015), depression (Yue et al., 2015) and insomnia (Zhou et al., 2016).

In the present work, we proposed to do multiple frequency band analysis (MFBA) of large scale intrinsic brain networks. Specifically, the ALFF/fALFF in frequency band of <0.1 Hz, slow-5 and slow-4 are characterized using the TCs of intrinsic brain networks. It is different with previously introduced MFBA which is based on the TCs of voxels or atlas-defined regions. Interpretations of the altered ALFF in some regions or clusters are hard because spatially distinct brain regions might belong to the same intrinsic brain networks (Zuo et al., 2010; Yeo et al., 2011). Moreover, the TC of intrinsic brain network is more robust than that of single voxel or regions to the disturbance of head motion, respiration and vascular pulsatility (Zuo et al., 2010). Except one study on power spectrum of TC of DMN in schizophrenia (Mingoia et al., 2013), to our best of knowledge, MFBA of large scale intrinsic brain networks has not been systemically investigated.

Schizotypal personality disorder (SPD) is the prototypical schizophrenia spectrum personality disorder, and shares abnormalities of gene, phenomenology and cognition with individuals with schizophrenia (Siever and Davis, 2004). SPD is usually characterized by delusion, no obvious hallucinations, peculiar thinking or behavior and lack of communication between people (Hur et al., 2016). According to the Diagnostic and Statistical Manual (DSM), the subjects with SPD are always suspicious, arrogant, and easily produce a sense of shame^1^. However, subjects with SPD have rarely been exposed to antipsychotic medications and hospitalization, which are inherent confounds to schizophrenia. These two features have made SPD the ideal model to reveal the core processes of schizophrenia (Rosell et al., 2014).

Comparing to numerous investigations using structural MRI and task-related fMRI, few studies have been conducted using rs-fMRI (Hazlett et al., 2012; Fervaha and Remington, 2013). Among the few studies, we have previously demonstrated the altered default mode network functional connectivity in SPD (Zhang et al., 2014), and Gerretsen et al. (2014) have found that increased connectivity in DMN, the self-referential network (SNR) and DAN. Overall, MFBA of large scale intrinsic brain networks in SPD has not been systemically investigated. Hence, we propose a hypothesis that the ALFF/fALFF in three frequency bands of <0.1 Hz, slow-5 and slow-4 in SPD are different with those in normal control groups.

## Materials and Methods

### Participants

Participants were 18 normal controls (NC; all male, average age: 20.3 ± 0.9 years, 19–22 years) and 18 patients (all male, average age: 19.7 ± 0.9 years, 18–21 years) who met the criteria for SPD with a full diagnostic structured interview for DSM-IV Personality Disorders (Millon and Davis, 1996). They were screened from 3000 freshmen of one university. All participants were evaluated by the Scale for the Assessment of Negative Symptoms (SANS) and the Scale for the Assessment of Positive Symptoms (SAPS; Andreasen, 1989). None of the healthy controls has history of neurological diseases, any substance dependance, or clinically significant head trauma. None of the SPD patients was previously hospitalized or prescribed antipsychotic medications. This study was approved by the Medical Ethics Committee of Affiliated Zhongshan Hospital of Dalian University and was in accordance with the 1964 Helsinki Declaration and its later amendments or comparable ethical standards. All subjects gave written informed consent in accordance with the Declaration of Helsinki.

### MRI Data Acquisition

All participants underwent structural and functional MRI scan using a three TMR scanner (Siemens, Verio, Germany) with one 8-channel head coil. The foam pads were used to fix the subjects’ head and prevent motion artifact. The T1-weighted images were acquired using a magnetization prepared rapid gradient echo (MPRAGE) sequence, and the protocol parameters were as follows: repetition time (TR) = 2300 ms; echo time (TE) = 3.0 ms; inversion time (TI) = 900 ms; flip angle = 9°; slice thickness = 1.0 mm; no interslice gap; 176 sagittal slices; matrix size = 256 × 256. In addition, T2-weighted images were also taken to exclude potential pathological alternations of the brain. The resting state fMRI was performed with a gradient-echo planar imaging (EPI) sequence. The specific EPI parameters are TR = 2000 ms, TE = 30 ms, flip angle = 90°, slice thickness = 4.0 mm, 1.0 mm interslice gap, 32 contiguous axial slices, matrix size = 64 × 64, field of view (FOV) = 240 × 240 mm^2^, 180 time points. Subjects were asked to relax and think of nothing in particular with eyes closed but were requested not to fall asleep. Wakefulness was confirmed immediately after the scanning session. The datasets generated for this study can be found in the repository of FigShare^2^.

### Multiple Frequency Band Analysis of Large Scale Intrinsic Brain Networks

**Figure 1:**
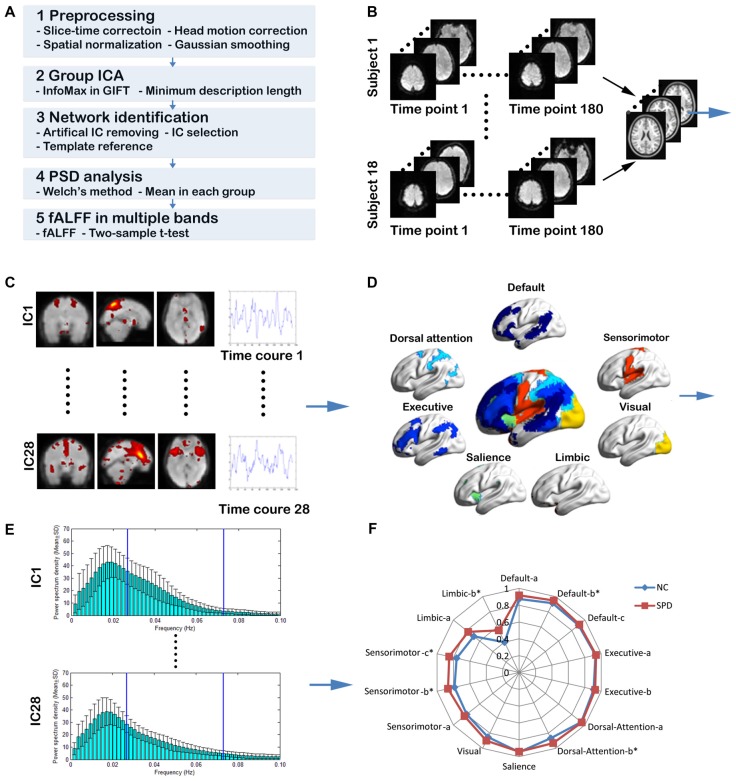
The schematic diagram for the procedures of multiple frequency band analysis (MFBA) of large scale intrinsic brain networks. **(A)** The overview of MFBA. **(B)** The rs-fMRI data is preprocessed through slice timing correction, motion correction, smoothing and normalization. **(C)** Spatial mapping of independent components (ICs) and time courses (TCs) are obtained through group independent component analysis (ICA). **(D)** Various large scale intrinsic brain networks are spatially constructed through combining some ICs. **(E)** The TCs are transformed into frequency domain using Welch method and divided into three bands: <0.1 Hz; slow-5; slow-4. **(F)** The fractional amplitude of low frequency fluctuations (fALFF) is calculated for each IC in three frequency bands and compared between normal control group and schizotypal personality disorder (SPD) group.

As illustrated in Figure 1A, the proposed MFBA of large scale intrinsic brain networks mainly consists of five steps. First, the rs-fMRI data is preprocessed through slice-timing correction, motion correction, normalization and smoothing. Second, the spatial mapping of independent components (ICs) and TCs are obtained through group ICA. Third, the large scale intrinsic brain networks are constructed through combining some ICs. Forth, the TCs are transformed into frequency domain and divided into three bands: <0.1 Hz; slow-5; slow-4. Fifth, the fALFF is calculated for each IC in three frequency bands and compared between NC group and SPD group. These five steps are represented schematically in Figures 1B–F, respectively. Detailed approaches are elucidated as follows.

#### Image Preprocessing

The first 10 volumes of each functional time series were removed, given that the initial MRI signals are unstable and participants need time to adapt the circumstances. The remaining 170 volumes (or time points) of images were analyzed. Subsequently slice-timing correction and head-motion correction (a least squares approach and a six-parameter spatial transformation) were performed. After EPI images were normalized to standard Montreal Neurological Institute space and resampled to 3 × 3 × 3 mm^3^, an isotropic Gaussian filter of 6 × 6 × 6 mm^3^ full width at half maximum (FWHM) was used to realize spatial smoothing. The data with head motion> 3.0 mm or 3.0° of maximal rotation were discarded. All the preprocessing was completed using Data Processing Assistant for Resting-State fMRI (DPARSF; Yan and Zang, 2010) based on Statistical Parametric Mapping (SPM8)^3^ and Resting-state fMRI Data Analysis Toolkit (REST^4^; Song et al., 2011).

#### Group Independent Component Analysis

The preprocessed data was decomposed into a set of independent components characterizing by the TCs and associated spatial maps, through the Infomax algorithm within the GIFT software^5^ (version 3.0a). Using minimum description length algorithm (Li et al., 2007), the number of ICs is estimated to be 28 for NC group and 29 for SPD group, respectively. Different numbers of ICs are resulted from the observation that the visual network appearing as one IC in NC group is divided into two ICs for SPD group.

#### Identification of Large Scale Intrinsic Brain Networks

For the IC selection, we first remove the ICs which have high spatial overlap with the vascular, ventricular, motion, and susceptibility artifacts (Allen et al., 2011). Second, to help evaluate the IC further, we calculate two measures from the spectra of TC of each IC: the dynamic range; the spectral power ratio of low frequency (<0.1 Hz) to high frequency (0.15–0.25 Hz; Rummel et al., 2013). The IC with higher dynamic range and spectral power ratio has high probability of being the one of large scale intrinsic brain networks, according to the expectation that TCs should be dominated by low frequency fluctuations. Fourteen ICs are determined as certain components of intrinsic networks and kept for further matching between NC and SPD groups and being assigned to different networks.

With the large scale intrinsic brain networks defined by Yeo et al. (2011) as the reference, seven intrinsic brain networks are constructed through combining some ICs identified by visual inspection. The seven networks are the DMN, executive control network, DAN, salience network, visual network, sensorimotor network and limbic network. After setting the Z-score threshold as 0.5 (i.e., the voxel with Z-score <0.5 is set a Z-score of zero), the spatial overlap ratios between corresponding ICs in NC and SPD groups are calculated. Meanwhile, the Pearson correlation coefficients between Z-scores of voxels within ICs of NC group and those of voxels within corresponding ICs of SPD group are obtained. According to the criteria of the maximal overlap ratio and correlation coefficient, each interested IC in NC group can find its counterpart in SPD group.

#### Power Spectral Density Analysis

The TC associated with each individual’s component assigned to various intrinsic brain networks is transformed from the time domain to the frequency domain through Welch’s (1967) method, as previously used by Mingoia et al. (2013). Specifically, the power spectrum is obtained by pwelch, a Matlab signal processing toolbox. Through dividing the input signal (170 points) into eight sections of equal length, each with 50% overlap, the power spectrum density with 129 bins of 0.0019 Hz, ranged from 0 Hz to 0.25 Hz, can be generated. As the examples shown in Figure 1E, the distributions of averaged power spectrum density in both NC and SPD groups are produced for further analysis.

#### ALFF/fALFF in Multiple Frequency Bands

ALFF and fALFF are calculated for each IC in three frequency bands, i.e., <0.1 Hz, slow-5 (0.01–0.027 Hz), slow-4 (0.027–0.073 Hz). Actually, fALFF is the power in corresponding band divided by the total power in the entire detectable frequency range (<0.25 Hz). Given the fALFFs of slow-3 band (0.073–0.198 Hz) and slow-2 (0.198–0.25 Hz) are very small and unreliable, we have not analyzed them. The central frequency and the width of the bands are defined according to the formula given by Buzsáki and Draguhn (2004). Specifically, the central frequencies of the bands follow a linear progression on a natural logarithmic scale with a constant ratio between neighboring frequencies, generating the separated frequency bands. Two-sample *t*-test is performed to examine if there is the significant difference between fALFF in NC and SPD groups (*p* < 0.05). For multiple comparisons, the false discovery rate (FDR) is controlled by the linear step-up procedure introduced by Benjamini and Hochberg (1995).

## Results

### Intrinsic Brain Network Constructed From Combination of Independent Components

**Figure 2:**
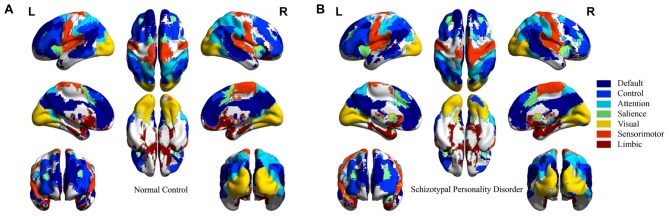
Seven large scale intrinsic brain networks constructed from spatial combination of 14 ICs for NC and SPD groups. **(A)** NC group. **(B)** SPD group.

Spatial distributions of seven networks are given in Figure 2 for NC and SPD groups. The details of how ICs combine to generate networks, the spatial overlap ratio (*r_so_*) and Z-score Pearson correlation coefficient (*r*_z_) between ICs in NC and SPD groups are presented in Table 1. Several observations can be gained. First, the spatial distributions of seven networks constructed from combination of ICs are consistent with those generated through the clustering analysis (Yeo et al., 2011). The quantitative comparison of results from two approaches is not accessible because one is volume-based and the other is surface-based. Second, most patterns of networks are convergent between NC and SPD groups. It origins from the fact that high *r*_so_ and *r*_z_ between ICs in NC and SPD groups. For most ICs, *r*_so_ ranges from 0.6151 to 0.7726; for sensorimotor-b and limbic-b, *r*_so_, not as high as other ICs, is only 0.4956 and 0.5638, partially due to the small volume of these two ICs. Except limbic-b and visual ICs, *r*_z_ is larger than 0.5861. Smaller *r*_z_ of visual IC might be resulted from that one visual IC is generated for NC group, however two ICs (left and right) are formed for SPD group. Third, the combination of different number of ICs or one single IC correspond to one large scale intrinsic brain network, indicating the divergence of scales and constructing approaches. The group ICA can generate fine-grained networks as an alternative to clustering algorithm. Forth, seven large scale intrinsic brain networks constructed from 14 ICs have covered most cerebral cortex areas for both NC and SPD groups. The generated atlas of each brain network can be used for further studies of spatial z-score distribution of functional connectivity, ReHo, and fALFF, functional network based on average time-series, and structural brain network based on DWI and tractography.

**Table 1:** The spatial overlap ratio (*r*_so_) and *Z*-score Pearson correlation coefficient (*r*_z_) between independent components (ICs) in NC and SPD groups.

Name of IC	Spatial overlap ratio (*z* > 1.0; *r*_so_)	*Z*-score Pearson correlation coefficient (*r*_z_)

Default-a	0.6608	0.7372
Default-b	0.7656	0.9043
Default-c	0.6627	0.5861
Executive-a	0.7449	0.7570
Executive-b	0.7086	0.8479
Dorsal-Attention-a	0.6166	0.8503
Dorsal-Attention-b	0.7327	0.7236
Salience	0.6430	0.6758
Visual	0.7109	0.3517
Sensorimotor-a	0.6151	0.8395
Sensorimotor-b	0.4956	0.3369
Sensorimotor-c	0.7726	0.8160
Limbic-a	0.5638	0.7543
Limbic-b	0.7331	0.8367

### Power Spectral Density of Large Scale Intrinsic Brain Networks

**Figure 3:**
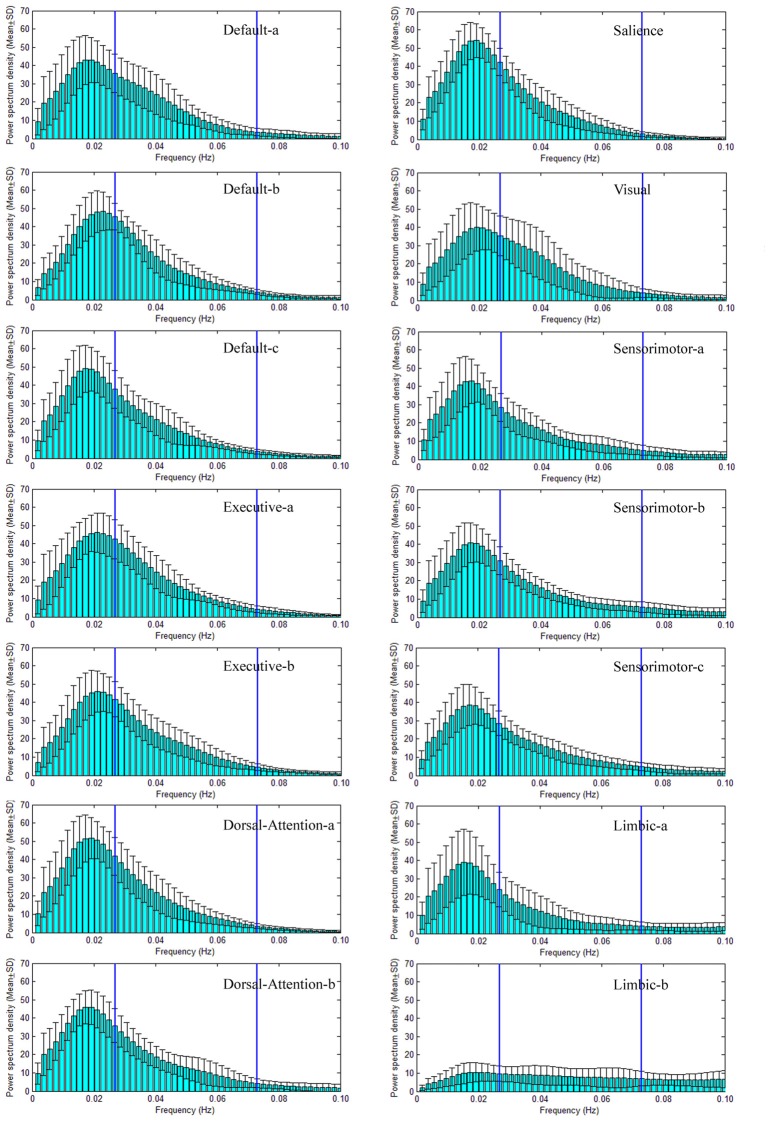
Power spectral density (PSD) of seven large scale intrinsic brain networks of NC group. There are seven large scale intrinsic brain networks spatially constructed by 14 ICs, and the PSD of the TC of each IC is presented. The default network consists of three ICs named Default-a, Default-b and Default-c (the name is just for convenience and does not indicate the order and meaning), the executive network includes two ICs (Executive-a and Executive-b), the dorsal attention network (DAN) has two ICs (Dorsal-Attention-a and Dorsal-Attention-b), the sensorimotor network comprises three ICs (Sensorimotor-a, Sensorimotor-b, Sensorimotor-c), the limbic network includes two ICs (Limbic-a and Limbic-b), both the salience and visual networks have one IC. The two vertical lines in each sub-figure of the PSD vs. frequency indicate the locations of 0.027 Hz and 0.073 Hz. Therefore, the frequency bands of slow-5 (0.01–0.027 Hz) and slow-4 (0.027–0.073 Hz) are marked through these two lines.

**Figure 4:**
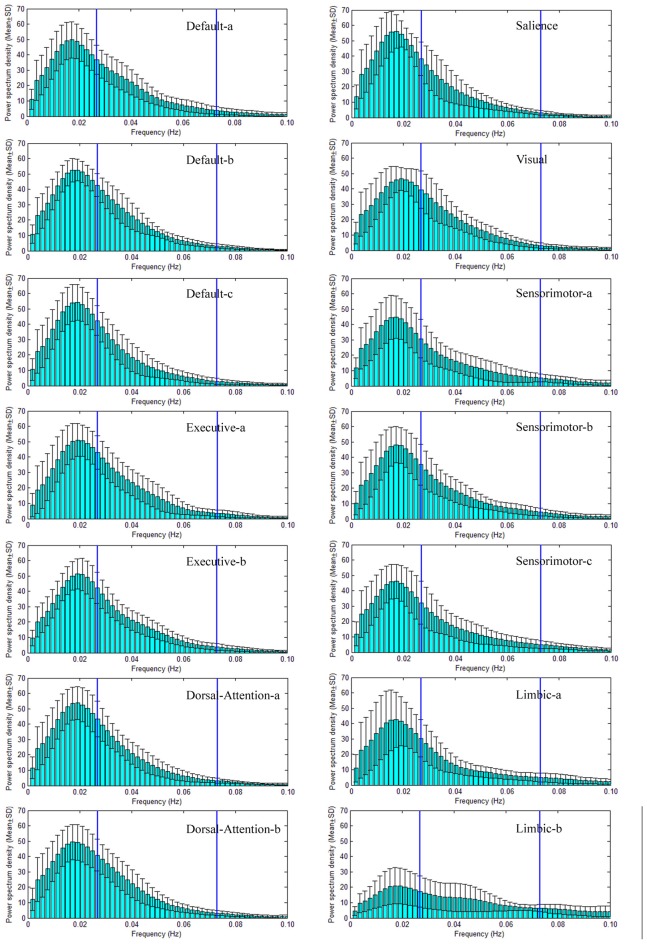
PSD of seven large scale intrinsic brain networks of SPD group. As same as to the NC groups, 14 ICs spatially form seven large scale intrinsic brain networks, and the PSD of the TC of each IC are given. The construction of each large scale intrinsic brain networks, the name and order of ICs are equal to those in NC group. The frequency bands of slow-5 (0.01–0.027 Hz) and slow-4 (0.027–0.073 Hz) are marked by two vertical lines in each sub-figure of the PSD vs. frequency.

Power spectral density (PSD) of large scale intrinsic brain networks has been given in Figures 3, 4 for NC and SPD group, respectively. The shapes of PSD look similar across both networks and groups though the extract amplitudes are different, i.e., the value increases gradually from frequency near zero, reaches a peak at about 0.02 Hz, and then decrease continuously with the frequency. It is in agreement with study of Damoiseaux et al. (2006). The peak of PSD decreases from the advanced cognitive network to primary sensorimotor networks with the maximum for the salience network. For limbic-b network, the lowest peak makes the curve of PSD quite different with those of the other networks. All the peaks are located in slow-5 band, which may partially explain the reason and necessity of dividing the frequency band into slow-5 and slow-4. Though the exact mechanism is unknown, the difference of PSD trend observed here presents different neural manifestations of slow-4 and slow-5, in line with studies at regional, interregional and network levels in time domain (Xue et al., 2014). The observation on the default modal network is consistent with those by Mingoia et al. (2013). Boyacioglu et al. (2013) also reported the differences of frequency spectra between advanced cognitive networks and primary cortex networks.

### fALFF in Multiple Frequency Bands

**Figure 5:**
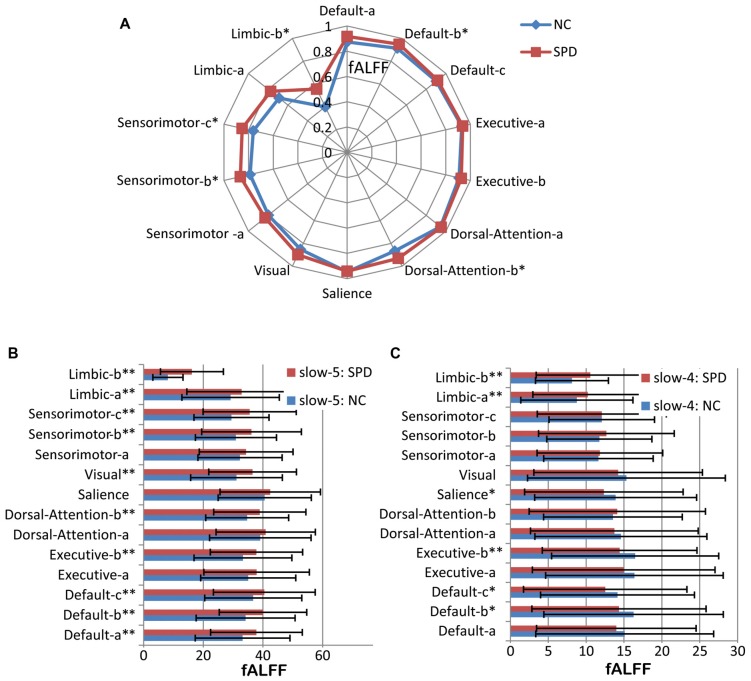
Fractional amplitude of low frequency fluctuation (fALFF) of large scale intrinsic brain networks in multiple frequency bands. **(A)** The frequency band of <0.1 Hz. **(B)** Slow-5 band. **(C)** Slow-4 band. One single asterisk indicates that there is a significant difference (*p* < 0.05) between SPD and NC groups, but it does not pass the false discovery rate (FDR) correction. Two asterisks mean that there is an FDR-corrected significant difference.

fALFF of three frequency bands for seven networks (14 ICs) and their comparisons between NC and SPD groups have been given in Figure 5. For the band of <0.1 Hz (Figure 5A), fALFF ranges 0.40–0.95 and 0.56–0.95 for NC and SPD groups, respectively. The advanced cognitive networks show higher fALFF (>0.87 for NC; >0.92 for SPD) than the primary sensorimotor networks and limbic networks for both groups. Thirteen of fourteen ICs (except the salience network) presents higher mean of fALFF for SPD group, five ICs have significant higher fALFF (*p* < 0.05) for SPD group. However, no one IC passed the FDR correction.

In the band of slow-5 (Figure 5B), some common features are found for the NC and SPD groups. First, the primary sensory and motor networks own lower fALFF than advanced cognitive networks (*p* < 0.05), which is in accordance with, but more significant than, the result in the band <0.1 Hz. It can be explained by the proposal that low frequency bands were associated with the integration of large-scale neural networks and long-distance connectivity, while high frequency bands correspond to local neural activity and short connections (Buzsáki and Draguhn, 2004). The cognitive networks belong to the association networks defined by connectivity between widely distributed regions, while sensorimotor networks are hallmarked by dense local connectivity to nearby areas (Buckner et al., 2013). Moreover, the salience network presents the highest fALFF in both groups.

Comparison of fALFF between NC and SPD groups demonstrates that all ICs in SPD have higher mean of fALFF than that in NC in slow-5 band, 10 of 14 ICs present statistical significance (*p* < 0.05, FDR corrected). It might suggest that there is wide spread disruption of functional brain organization in SPD, just as in early-onset Alzheimer’s disease (Adriaanse et al., 2014). Specifically, the function of advanced cognitive networks is overactive and the function of primary sensory networks is inhabited or disrupted.

For the band of slow-4 shown in Figure 5C, opposite trend is found. For SPD group, seven of eight ICs in advanced cognitive networks (except IC of dorsal-attention-b) display lower mean fALFF than that in NC, four presents statistic significant (*p* < 0.05), but only Executive-b passes the FDR correction. However, the mean of fALFF in sensorimotor network is still higher for SPD group though no significance exists, and the fALFF in SPD is significantly higher than that in NC. It might suggest that fALFF in SPD will be lower than that in NC for much higher frequency band (>0.073 Hz). Moreover, fALFF is larger in slow-5 band than in slow-4 band in all studied networks. It accords with previous observation of greater fALFF in slow-5 for the cortical areas but in slow-4 for the subcortical areas (Zuo et al., 2010; Wang Z. et al., 2014).

### ALFF in Multiple Frequency Bands

**Figure 6:**
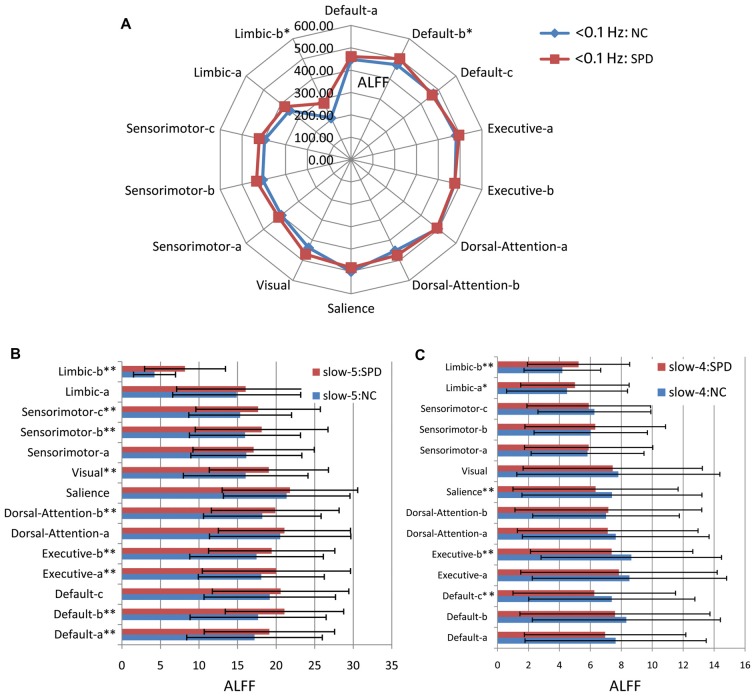
Amplitude of low frequency fluctuation (ALFF) of large scale intrinsic brain networks in multiple frequency bands. **(A)** The frequency band of <0.1 Hz. **(B)** Slow-5 band. **(C)** Slow-4 band. One single asterisk indicates that there is a significant difference (*p* < 0.05) between SPD and NC groups, but it does not pass the FDR correction. Two asterisks mean that there is an FDR-corrected significant difference.

Figure 6 gives the results of ALFF. Comparing Figures 5, 6, one can find that most results from ALFF and fALFF are consistent, except three slight differences. As shown in Figure 6A, ALFF in <0.1 Hz band has no significant difference between SPD and NC groups for Dorsal-Attention-b, Sensorimotor-c and Sensorimotor-b. For ALFF in slow-5 band (Figure 6B), Default-c does not pass the FDR correction (*p* < 0.05). No significant difference between SPD and NC is observed for ALFF of Default-b in slow-4 band (Figure 6C). These observations are supported by previous report that the measure of fALFF has higher specificity for it suppresses the physiological noise, while ALFF owns higher test-retest reliability (Zuo et al., 2010).

## Discussion

In the current study, we proposed a novel framework of conducting multiple frequency bands analysis (MFBA) to large scale intrinsic brain networks. Using this framework, we identified the default, executive, salience, dorsal attention, sensorimotor, visual and limbic networks in both NC and SPD groups, and examined their similarities and differences in spatial distributions, PSD, fALFF in three frequency bands (<0.1 Hz, slow-5 and slow-4). Overall, the spatial patterns and PSD distributions of networks are convergent between NC and SPD groups. The PSD and fALFF are different across networks, frequency bands and groups. It is suggested that the proposed MFBA method is very powerful to characterize the different networks and neurological disorders. Neural activity in SPD has been disrupted globally, indicated aberrant fALFF of various networks in different frequency bands.

The advanced cognitive networks display higher fALFF than the primary sensorimotor networks in three frequency bands. It might be explained by the connectivity hallmarks of these two categories of networks. The advanced cognitive networks correspond to association networks which are defined by long connectivity between widely distributed areas, whereas the primary sensorimotor networks are distinctively characterized as dense local connectivity to nearby cortex (Power et al., 2011; Yeo et al., 2011; Buckner et al., 2013; Glasser et al., 2016). Meanwhile, Buzsáki and Draguhn (2004) suggested that lower frequency bands are associated with the integration of large-scale neural networks, while higher frequency bands correspond to local neural activity.

We found greater fALFF in the slow-5 band than in the slow-4 for all investigated networks. This finding can still be explained by the suggestion of Buzsáki and Draguhn (2004) given they are located in cerebral cortex. It accords with previous observations, and only subcortical regions contributing to local events present great fALFF in slow-4 than in slow-5 (Yu et al., 2014).

### Large Scale Intrinsic Brain Networks

It is worth noting that the combination of several components into a network or assignment of one single component to a specific network is based on visual inspection with the reference of the coarse (7-network) parcellations by Yeo et al. (2011). Therefore, neither may our estimated networks exactly match with those in the previous literature, nor the assigned name of network solely corresponds to its functions.

For example, default network defined in current study is composed of three ICs, and covers the medial prefrontal cortex (PFC), the posterior cingulated/retrosplenial cortex, the inferior parietal lobule (IPL) and the medial temporal lobes. However, previous studies usually considered DMN to be separated into anterior and posterior regions (Damoiseaux et al., 2006; Calhoun et al., 2008; Zhang et al., 2014), though the main regions are overlapped in previous and our studies. The heuristic reference label of the salience network is adopted from Seeley et al. (2007) and Buckner et al. (2013), it is referred as (or closely adjacent to) ventral attention network (Yeo et al., 2011) or cingulo-opercular network (Dosenbach et al., 2007). It mainly consists of anterior cingulate cortex (ACC) and dorsal anterior insular cortex (dAIC; Seeley et al., 2007; Uddin, 2015). The DAN includes dorsolateral PFC, FEFs, inferior precentral sulcus, superior occipital gyrus, middle temporal motion complex, and superior parietal lobule (Corbetta and Shulman, 2002; Fox et al., 2005). The executive control network is also referred as frontoparietal control network (Yeo et al., 2011), or control network (Buckner et al., 2013), consisting of lateral PFC, precuneus, the anterior extent of IPL, medial superior cortex and the anterior insula (Vincent et al., 2008; Spreng et al., 2010). Actually in previous studies, the frontoparietal control network also includes anterior insula which is one part of salience network. In the work by Seeley et al. (2007), it includes dorsolateral PFC (DLPFC), lateral parietal cortex, dorsomedial frontal/pre-SMA and ventrolateral PFC. To be consistent with the results by Yeo et al. (2011), we combined three distinct ICs into the sensorimotor network. Actually, it covers the regions for the motor, somatosensory and auditory functions, and the component-c corresponds to the auditory regions. Limbic networks are not well defined in the present work. After assigning several large ICs into above six networks, we put the remaining comparatively large ICs into the so-called limbic networks.

It is important to know the functions of DMN, CEN, DAN, salience network and limbic system because it helps interpret the disrupted brain functions in SPD from the altered fALFF/ALFF in multiple frequency bands (see “Methodology Advantage and Significance” section). DMN is associated with self-oriented and social cognition (Buckner et al., 2008; Sridharan et al., 2008; Andrews-Hanna et al., 2014). The executive control network is to operate on identified salience (Seeley et al., 2007), and is involved in the maintenance and manipulation of information, as well as decision making. The salience network takes central role in the detection of behaviorally relevant stimuli and the coordination of neural resources from DMN (internally directed action) and CEN (externally directed action; Uddin, 2015). DAN subserves externally directed cognition (Spreng et al., 2013). Limbic system is involved in motivation, emotion, learning and memory.

### Methodology Advantage and Significance

In the proposed MFBA framework, the large scale intrinsic brain networks are constructed from spatially ICs resulted from group ICA at first. Second, the TC of each IC is transformed into frequency domain to characterize its PSD. Third, the fractional amplitude of LFO (i.e., fALFF) in multiple frequency bands (<0.1 Hz, slow-5, and slow-4) can be determined. We used Welch method to estimate PSD, which is more robust than peroidogram methods employed in previous study (Zuo et al., 2010). Other methods such as wavelet-based method and Chronux spectral analysis are also possible (van Vugt et al., 2007; Duff et al., 2008).

Only one ALFF measure is not enough to sensitively distinguish the alteration of low and high frequency bands, which correspond to different oscillators and connectivity (short or long, local or global, for segregation or for integration). Alternations of ALFF based spontaneous BOLD signal are frequency dependent or specified (Zuo et al., 2010; Wang Z. et al., 2014; Yu et al., 2014). MFBA is more sensitive than ALFF analysis. For example, the schizophrenia group exhibited significantly higher spectral power than controls at a frequency bin 0.0797 Hz and 0.0858 Hz (Mingoia et al., 2013). Similar to ALFF, functional connectivity and its alternations are also frequency-specific (Salvador et al., 2008; Xue et al., 2014).

Actually, we do MFBA for TC resulted from the group ICA, not for TC of each voxel. The advantage lies in the ability of reducing the dimensionality of data, facilitating comparisons, enhancing SNR and so on, similar to brain parcellation based analysis (Glasser et al., 2016). Time series after pre-processing of each voxel were input into PSD toolbox to calculate amplitude of LFOs and fALFF. The resulting ALFF and fALFF were converted into Z-scores and the corresponding z-score map within gray matter mask is generated to further group-level analysis. The voxel-wise analysis of time is local and sensitive to motion (head, inspiration, etc.), the influence of large vessels. After volume and artificial analysis based on (LF to HF power ratio) vs. (Dynamic range) (Smith et al., 2013) and visual inspection with known references, ALFF and fALFF of TCs can avoid mentioned disturbances to voxel-wise analysis. Using ICs to construct large-scale intrinsic brain networks owns apparent advantages. ICs is data-driven approach to obtain potential functional networks with spatially distributed brain regions. To combine various ICs into advanced cognitive networks is helpful to build up hierarchical brain networks. It also helps understand architecture of cognitive networks, their components (or sub-network) and functions.

As per our previous study (Zhang et al., 2014), we performed the group ICA in the segregate two groups. This method originates from the hypothesis that the health controls and SPD patients are two different groups, their combination will increase the heterogeneity of data, and the group ICA results from this combined group might not characterize the spatial and temporal features of SPD. To evaluate the matching degree between the same IC from two groups, the spatial overlap ratio (*r*_so_) and Z-score Pearson correlation coefficient (*r*_z_) are analyzed (as shown in Table 1). It is shown that most patterns of networks are convergent between NC and SPD groups (high *r*_so_ and *r*_z_). However, the ICs from two groups are not completely overlapped spatially, which might be related to the disturbance of BOLD signal of SPD. The spatial difference of the same IC does not influence their comparison of measures in temporal or frequency domain (i.e., fALFF). In the study by Anderson and Cohen (2013), the individualized ICA had been implemented, based upon the hypothesis that the different networks operate between schizophrenia patients and health controls. In summary, there is no consensus regarding the way of ICA, in the segregate groups or combined group.

Wu used the template by Yeo et al. (2011) generated from the resting state fMRI data (or the intrinsic functional connectivity) of 1000 subjects by a clustering approach. This template is surface-based, which is thought to more accurate than the volume-based templates in registration (Qi et al., 2015). The ICA template generated from rs-fMRI data of 603 healthy subjects (Allen et al., 2011) and another available template from FIND laboratory (Shirer et al., 2012) containing 90 ROIs across the 14 Independent Component Networks (ICNs) can also be used in future.

Recently, the low frequency steady-state brain response has been studied by the MFBA (Wang Y. et al., 2014; Wang et al., 2015, 2018; Wang Y. et al., 2016). It is demonstrated that the cognitive activities can change the power at different frequencies. In our study, we use the MFBA to reveal alternations of fALFF in different networks in SPD patients in a “resting” state. Compared with the case of cognitive activity, the “resting” state can be considered as a baseline.

To the best of our knowledge, multi-band fALFF of large-scale intrinsic brain networks constructed through group ICA has not been studied, for both the healthy control and SPD.

### Disruptive Large Scale Intrinsic Networks in SPD

Our new finding on SPD is that higher fALFF in the frequency bands of <0.1 Hz in SPD group for most networks compared with NC group, and this trend becomes more pronounced in slow-5 band. This finding indicates there is a global disruption in the organization of SPD brain networks, similar to the observation that all three networks (SN, DMN, CAN) of patients with schizophrenia show structural and functional deficits (Palaniyappan et al., 2011). It can be explained by the interaction of networks which result in the spreading from the primary deficit (Menon, 2011). In line with our results, Yu et al. (2014) also reported altered fALFF in widespread brain areas. However, which network or brain region is the primary deficit is unknown and worthy of further study. Causality analysis of connectivity between these networks might be a promising avenue (Friston, 2009) as what have been done in schizophrenia (Palaniyappan et al., 2013).

Higher fALFF is the manifestation of abnormal spontaneous neural activity. In different networks, it may show distinct representations and give rise to different symptoms for SPD. Higher fALFF in DMN means there might be a DMN suppression deficit in SPD. Lack of DMN suppression has been reported in a growing body of work in schizophrenia patients and their first-degree relatives (Pomarol-Clotet et al., 2008; Whitfield-Gabrieli et al., 2009; Gerretsen et al., 2014). Given the idea that DMN suppression functionally correlates to goal-directed cognition (Menon, 2011; Anticevic et al., 2012), lack of suppression will result in bad performance in cognitively demanding task, explaining the symptom of cognitive impairment associated with SPD. Moreover, aberrant neural activity in DMN in SPD might lead to deviant functional connectivity, and then positive symptoms like hallucination and delusions as observed in schizophrenia patients (Rotarskajagiela et al., 2010; Woodward et al., 2011).

In CEN and DAN, higher fALFF in slow-5 and lower fALFF in slow-4 for SPD suggest that local connectivity in these networks corresponding to fALFF in slow-4 is impaired or certain region is broken. In other words, functional segregation in these networks is reduced, whereas the functional integration is enhanced. Though this hypothesis needs the more direct evidences from graph theory analysis on intrinsic connectivity network (in preparation), the decreased network connectivity and efficiency of CEN networks have also been reported in schizophrenia (Bassett et al., 2008). Considering the function of CEN, the current finding appears to be associated with the SPD symptoms of the working memory deficits and disorganization, similar to the finding in schizophrenia (Menon, 2011; Woodward et al., 2011). The abnormal fALFF in CEN might be associated with deficits of attention in SPD (Luck and Gold, 2008).

For the salience network, no significant difference of fALFF is observed in band of <0.1 Hz and slow-4 band. However, we observed the functional connectivity between salience network and three DMN components, one CEN component, and one dorsal attention component significantly decreases in SPD (in preparation). It is in agreement with the observations that the causal influence from the salience network (right AIC) on the DMN and CEN in schizophrenia turns weak (Palaniyappan et al., 2013; Manoliu et al., 2014). A growing body of literature has indicated that the structural and functional abnormality of salience network is a key neuropathological component in schizophrenia. The increased fALFF in frontal regions is because these regions are dysregulated in the context of working memory task performance. Dysregulation of medial frontal regions is associated with self-directed thoughts, with the consequence that the source of internal and external stimuli could become confused, which may provide a neurophysiological basis for hallucinations (Palaniyappan et al., 2013; Manoliu et al., 2014).

In primary cortex, the visual, sensorimotor and auditory networks present higher fALFF in slow-5 band for SPD group. In slow-4 band, the statistical significance does not exist for three sensorimotor networks, even the visual network in SPD group displays lower mean fALFF. Given the primary networks are characterized as the local connectivity and specialized function, fALFF in high frequency band was to be dominated. The reduced fALFF in high frequency might partially explain the disruptions of these networks. Compatible with the present finding, the reduced activation in visual networks in SPD has been observed in several previous studies (Camchong et al., 2008; Aichert et al., 2012; Meyhöfer et al., 2015), and lower activation in sensorimotor networks has been reported (Hong et al., 2005; Keedy et al., 2006).

In limbic system, the increased fALFF in low frequency band (slow-5 and slow-4) becomes more significant, corresponding to the reduced fALFF (>0.1 Hz) in high frequency. Since limbic system includes some subcortical structures of the hippocampus, anterior groups of thalamic nuclei, hypothalamus, mammillary body, it is supposed to be oscillation of high frequency (Salvador et al., 2008; Zuo et al., 2010). Deficits in limbic system characterized as reduced fALFF in high frequency might be implicated in symptoms of SPD. For example, dysfunction in the frontal-striatal-thalamic circuitry, the neural circuitry involved in rewarding has been reported in SPD (Hazlett et al., 2008; Hur et al., 2016) and schizophrenia (Hoptman et al., 2010). Deficits in reward processing and/or stimulus saliency, dulling of emotional expression might relate to the alternation in the right striated region. Moreover, the volume of subcortical structures seems to be reduced in SPD, hippocampus is the key node of pathology (Fervaha and Remington, 2013).

The signal from lower frequency bands is associated with the integration of large-scale neural networks and long-distance connectivity, which may be primarily mediated by cortical regions, especially the brain’ hub nodes, such as the mPFC and IPL. Conversely, higher frequency signals have been linked with more local neural activity and short connections, which maybe largely constituted by the more primitive subcortical regions. That phenomenon may provide an explanation for our findings of greater LFO amplitudes in the cortical areas in slow-5 band but greater amplitudes in the subcortical areas in relatively higher slow-4 band (Wang Z. et al., 2014).

### Limitations and Future Works

While determining spatial locations of each IC, one threshold of Z > 0.5 is employed to the Z-map of IC, indicating that the voxels with Z < 0.5 will be set zero and excluded from the IC. This threshold is roughly decided according to the tradeoff that overlapped regions between different ICs are minimized and the sum of ICs occupy most gray matter volume. It is unknown if this threshold is applicable to other studies. To increase this threshold will make *r_so_* and *r*_z_ increase.

According to the theoretical scale-free model, the peak of curve between the power and frequency should be located in the lower frequency bands. Our results and previous studies (Mingoia et al., 2013) showed the peak is located in slow-5 band for the data is not long enough (only 170 points). This issue can be addressed in the future study through extending the rs-fMRI scanning time.

The TC of intrinsic brain network is more robust than that of single voxel or regions to various noises (Zuo et al., 2010). TC of each individual’s IC will suppress high-frequency components for the neural activity of the short-range connections. However, if one region has a good homogeneity of neural activity, the suppression to high-frequency components will be limited. That is the reason why the group ICA is performance twice (for healthy control and SPD patients). The final goal is to extract the IC with homogeneous neural activity.

Intrinsic functional connectivity provides a powerful and unique tool to provide insight into human brain organization. However, fcMRI is based on an inherently ambiguous measure that reflects constraints both from static anatomical connectivity and from poorly understood functional coupling changes that are dynamic. For this reason, fcMRI is best used to a tool for generating hypothesis about brain organization that will require further study with external methods (Buckner et al., 2013).

Actually, the generated volume-based atlas or parcellations of each brain network is consistent with previous studies (Allen et al., 2011; Yeo et al., 2011; Shirer et al., 2012) by visual inspection. We are confident of using this atlas in the current SPD data to study the ReHo and fALFF of these large-scale intrinsic brain networks, to construct the functional whole brain network using time-series of independent components, and to build up the structural brain network using DWI data (we had scanned) and tractography algorithms.

The neural mechanism of several frequency bands such as Alpha, Beta, Theta and Gamma might be known (Buzsáki et al., 2013). For example, the Theta oscillations (4–10 Hz) are supported by intracellular and circuit characteristics of the septo-hippocampal-entorhinal system. The LFOs (0.01–0.1 Hz) reflect the periodic modulation of gross cortical excitability and the long-distance synchronization of neurons. However, the neural mechanism of slow-5 and slow-4 is not clear, though we know the principle that low frequency bands are associated with the integration of large-scale networks and high frequency bands correspond to local neural activity (Buzsáki and Draguhn, 2004). To use the steady-state BOLD responses to modulate low frequency neural oscillations and perform MFBA might help clarify the neural mechanism in the future study (Wang Y. et al., 2014).

## Conclusion

The present work has demonstrated that the proposed MFBA method can characterize the large scale intrinsic brain networks through calculating the PSD and ALFF/fALFF of TC resulted from group ICA of rs-fMRI data. The PSD and ALFF/fALFF are different across networks, frequency bands and subject groups (NC or SPD). Intrinsic brain networks with different connectivity (short or long) and functions (segregation or integration) might correspond to oscillators with different frequencies and therefore present different characteristics in each frequency band. The proposed MFBA methods are proved to enable revealing the frequency-dependent alternation of ALFF/fALFF for seven networks in SPD, which may help interpret the neuropathology disruptions in SPD and correlate them with behavioral symptoms.

## Author Contributions

SQ, YS and JW designed and directed the study. JS, YS and JW recruited participants and acquired data. SQ, QG and XX analyzed the data. SQ and YT drafted the manuscript. All authors revised and approved the final version of the article.

## Conflict of Interest Statement

The authors declare that the research was conducted in the absence of any commercial or financial relationships that could be construed as a potential conflict of interest.

## Footnotes

^1^https://www.psychiatry.org

^2^https://figshare.com/s/3e4f9be7266f61e6c74a

^3^http://www.fil.ion.ucl.ac.uk/spm

^4^http://www.restfmri.net

^5^http://mialab.mrn.org/software/gift/index.html
